# Clinical Beneficial Effects of Using Crystalloid only in Recipients of Living Donor Liver Transplantation

**DOI:** 10.3390/ijerph14111418

**Published:** 2017-11-20

**Authors:** Chia-Jung Huang, Kwok-Wai Cheng, Chao-Long Chen, Shao-Chun Wu, Tsung-Hsiao Shih, Sheng-Chun Yang, Sin-Ei Juang, Ying-En Lee, Chiu-En Huang, Bruno Jawan, Chih-Hsien Wang

**Affiliations:** 1 Department of Anesthesiology, Kaohsiung Chang Gung Memorial Hospital, Chang Gung University College of Medicine, Kaohsiung 83301, Taiwan; moamd@hotmail.com (C.-J.H.); kwcheng@cgmh.org.tw (K.-W.C.); shaochunwu@gmail.com (S.-C.W.); 8802057@cgmh.org.tw (T.-H.S.); eternity0602@hotmail.com (S.-C.Y.); juangsinei@gmail.com (S.-E.J.); en0718@cgmh.org.tw (Y.-E.L.); joanne92@cgmh.org.tw (C.-E.H.); jawanb@hotmail.com (B.J.); 2 Department of Surgery and Liver Transplant Program, Kaohsiung Chang Gung Memorial Hospital, Chang Gung University College of Medicine, Kaohsiung 83301, Taiwan; clchen@cgmh.org.tw

**Keywords:** anesthesia, 5% albumin, blood product, crystalloids, living donor liver transplantation

## Abstract

*Objective*: Liver transplantation (LT) is a major surgery associated with intraoperative massive fluid shift, which is usually replaced by crystalloid, 5% albumin (colloid) and blood products. We studied 15 patients from 477 consecutive recipients of adult living donor liver transplantation. Each patient received crystalloid only during LT. Whether LT provides any clinical benefit is not clear and must be determined. *Methods and Patients*: The anesthesia records of 477 adult LDLT were reviewed retrospectively. The patients were divided into three groups according to the fluids received. Group I (GI) had received blood products, 5% albumin and crystalloid, group II (GII) received 5% albumin and crystalloid, and group III (GIII) received crystalloid only. The characteristic intraoperative variable and postoperative acute rejection and survival rate were compared amongst groups by using One Way ANOVA post hoc with Bonferroni and by Ficher’s Exact test and Chi-square χ^2^ test. *Results and Conclusions*: GIII had less intraoperative ascites and blood loss; they also had more stable hemodynamics. Furthermore, they could be extubated significantly earlier than GI, and the one- and three-year survival rates were excellent, with 100% in GIII, while that of GI and GII were 94.1%, 90.5% and 98.6%, 94.5%, respectively.

## 1. Introduction

Liver transplantation (LT) is a major surgery associated with intraoperative massive fluid shift which should be appropriately replaced with crystalloid, 5% albumin (colloid) and blood products as needed to maintain acceptable hemodynamics, ensuring sufficient blood supply to vital organs. Unstable hemodynamics are common in LTs due to blood loss and surgical intervention or manipulation of the large vessels, such as clamping and unclamping of the portal vein and inferior vena cava (IVC) [1]. Crystalloid with additional colloid with or without blood products are usually given intraoperatively [2,3,4]. In our data of 477 consecutive recipients of adult living donor liver transplantations we have retrospectively collected 15 patients, in which the living donor liver transplantation (LDLT) was performed with crystalloid, without 5% albumin or any blood products. Since 5% albumin is known to bind hormones, drugs, mineral ions and bilirubin [5] and blood products, transfusion may transmit several kinds of infectious diseases and modulate the immunology aspect of the recipient, inducing malignant tumor recurrence and transfusion-associated graft-versus-host disease [6]. Whether infusion of pure crystalloid only, without 5% albumin and blood products, in LDLTs has any potential preoperative and postoperative clinical benefits is not clear. The aim of this study is to analyze and compare the characteristics of these patients and their intraoperative hemodynamic changes and postoperative outcomes with other LDLTs requiring additional 5% albumin and blood products. The clinical information suggesting that LDLTs can be performed with pure crystalloid only is probably valuable, particularly for patients with religious objections to blood transfusion [7]. 

## 2. Method and Patients

The common practice for fluid management in our LT program was reported previously [3,8]. In brief, all the patients received initially only crystalloid as maintenance fluid and replaced the initial blood loss with a volume of three- to four-fold the total blood loss, 5% albumin replacement was used in the event of unstable hemodynamic status, and blood product(s) transfusion were given for blood loss of >20% of the total blood volume or the hemoglobin (Hb) at 6–7 g/dL and hematocrit (Hct) at 20%. The end target of blood transfusion was set at 8–9 g/dL after transfusion [3,9].

Dopamine at 2 µg/Kg/min was used throughout the operation to support the renal function and maintain a urine output >1 mL/kg/h. The surgical procedure performed without using a veno-venous bypass. No prophylaxis correction of abnormal coagulation data, and no autotransfusion, blood salvage and antifibrinolytics were used. Arterial blood gases (ABG) were measured at least 5 times during the LDLT: after induction of the anaesthesia; 2 and 4 h after skin incision; at the anhepatic phase; 5–10 min after reperfusion; and at the end of the operation using a blood gas machine (Ciba-Corning, 288 Blood Gas System, Ciba Corning Diagnostics Corp., Metfield, MA, USA). Five to ten mg/kg of calcium chloride or equivalent of calcium gluconate were given to maintain serum Ca^++^ level at about 1.0 mmol/L [10]. Sodium bicarbonate was given to buffer the metabolic acidosis when the base excess was >−5.

Approval for this retrospective study was obtained (99–3249 B) from the Institutional Review Board of our hospital. The anaesthesia records of all adult LDLTs performed consecutively between June 1994 and June 2011 were reviewed retrospectively. Pediatric living donor liver transplantations (younger than 18 years old) and all cadaveric liver transplantations were excluded. The patients’ information was anonymized and de-identified prior to analysis. The characteristics of intraoperative fluid status and management including volume of blood loss and ascites drainage, amount of leukocyte-poor red blood cells, fresh frozen plasma (FFP), and 5% albumin and crystalloid given were accurately recorded [11]. Other parameters including patient characteristics, duration of anaesthesia, pre- and postoperative hemoglobin (Hb), platelet count, international normalized ratio (INR), APTT and serum albumin were also collected. Patients were grouped based on the type of infusion and transfusion received into: group I (GI) crystalloid, blood products with 5% albumin (Biotest Pharma GmbH, Dreieich, Germany); group II (GII) 5% albumin; and crystalloid, group III (GIII) crystalloid only. 

Patients’ characteristics and intraoperative variables were compared using One Way ANOVA post hoc with Bonferroni. Postoperative acute rejection of the graft, proved by liver biopsy, within the first year and survival rate for one and three years were analyzed and compared by Ficher’s Exact test and Chi-square χ^2^ test. All data was given as mean ± standard deviation and a *p* value < 0.05 was regarded as significant.

## 3. Results

A total of 477 adult patients with complete anesthesia records were reviewed retrospectively. Patient characteristics were presented in Table 1. 

**Figure 1:**
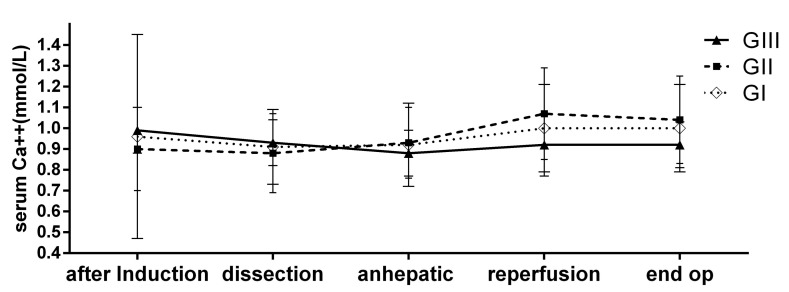
The changes of serum Ca^+ +^ among three groups were not significant difference.

There were 389 patients who received blood products with 5% albumin and crystalloids (GI), 73 who received 5% albumin and crystalloids but no blood products (GII), and 15 received crystalloids only without blood products or 5% albumin (GIII). There were no significant differences among the three groups for age, weight, the duration of anesthesia, INR, APTT, and the intraoperative ionized calcium level, which was maintained at the lower normal limits (Figure 1 and Table 1). Platelet count of GI and GIII were similar, while that of GII was significantly better than that of GI and GIII (Table 1). Hb of GI was significantly lower before operation than that of GII and GIII; at the end of the operation the Hb level of GI remained significantly lower than the other two groups with a value of 9.1 ± 1.5 g/dL.

Figure 1 shows the changes of the serum ionized calcium level during the LDLTs of the three groups. The levels could be maintained at lower normal limits without significant difference amongst groups after induction of the anesthesia, during dissection phase, anhepatic and reperfusion phase as well as at the end of the surgery. 

However, Table 1 revealed that calcium replacement was significantly different for each group. GI required the most with 52.1 ± 59.2 mEq, while an intermediate dose was provided for GII with 28.0 ± 11.4 mEq and GIII required the smallest dose with 9.15 ± 6.3 mEq. Patients of GI had experienced significantly more intraoperative ascites and more blood loss during the procedure in comparison to those of GII and GIII. Consequentially, patients of GI also received significantly more blood products including PRBC, FFP, platelet, 5% albumin and crystalloids in comparison to the other two groups (Table 1). As a result, GI patients received significantly more sodium bicarbonate for the maintenance of an acceptable pH, as more episodes of intraoperative metabolic acidosis occurred compared to GII and GIII. The duration for postoperative ventilation was also significantly longer in GI compared to GII and GIII. 

Table 2 reveals that the one-year acute rejection of the liver graft proved by liver biopsy was significantly different with a rate of 20.4%, 38.4% and 14.3% for GI, GII and GIII, respectively. The one- and three-year survival rates for GI, GII and GIII were 94.1%, 98.6%, 100% and 90.3%, 94.5% and 100%, respectively. 

## 4. Discussion

Table 1 revealed that the blood loss of our adult LDLT varied from minimal to massive; indeed, the amount of blood loss is the determinant factor which decided what kind of fluids the patients had received. Minimizing the surgical bleeding by improving the surgical technique and anesthesia management [3,12], aiming to reduce blood transfusion, or even foregoing blood transfusion is always the goal of all participants of the transplant team. Fluid restriction during dissection phase and forced diuresis with low doses of dopamine and furosemide were applied to keep the central venous pressure low [12]. Table 1 shows that LDLT could be performed without blood transfusion but with 5% albumin and crystalloid (GII) when the blood loss could be kept lower than 700 mL, and when the blood loss was less than 500 mL, the procedure could be done with crystalloid only (GIII). We had previously reported a pediatric case, in which the patient only received crystalloids without 5% albumin or blood transfusion, so that the patient experienced stable hemodynamic status with less metabolic acidosis and needed less supplemental calcium during LDLT compared to other patients who received both 5% albumin and crystalloids [10,13]. Patients’ characteristics of the current study and the surgical outcome of the crystalloid group (GIII) had indeed provided some beneficial information in adult LDLT setting. Table 1 and Figure 1 show that the serum ionized calcium level amongst groups were similar, but the amount of calcium replacement needed differed significantly amongst the three groups in preventing ionized hypocalcemia (IH); GI required greater than GII, and GIII required the least. The underlying mechanism could be due to the fact that both citrate [14,15] and 5% albumin [10,16] bind the free ion calcium. Citrate, a routine anticoagulant for blood bank products, binds to calcium ion and disrupts the blood clotting system, and is metabolized in the liver [17]. Routine calcium replacement for blood transfusion is not recommended [18] but in the case of LT for end stage liver disease, the risk of IH originated not only from massive blood product transfusion at a rapid rate but also resulted from insufficient liver metabolism of the exogenous citrate [16,19], of which the citrate level could indeed increase to 20 to 100 times higher in LT compared to other situations, reaching the toxic level that may lead to citrate intoxication with possible lethal consequences if not corrected [20]. Furthermore, the commercially available 5% albumin preparation is not only known to bind drugs, hormones, and bilirubin, but it also binds mineral ions including ionized calcium [5]. Infusion of albumin to treat hypoproteinemia in adults [16] as well as in neonates [17] causes a significant decrease in serum Ca^++^. Similar findings were also found in fluid resuscitation of patients with severe trauma [19]. The use of colloids was associated with a significant decrease of ionized calcium but this side effect was not found in crystalloids, which implied that the crystalloid solution did not bind free calcium ions, and thus the risk of IH was relatively low in comparison to using citrate-contained blood products and 5% albumin. It is very hard to plan or predict on whom the LT anesthesia can be performed with crystalloid only. Even though only 3.1% of patients in our series could be managed by crystalloids only, we have shown that fluid management is critical and can feasibly reduce the risk of IH to minimal. Table 1 also showed that GIII patients required less sodium bicarbonate, indicating that patients experienced a more stable acid base balance and hemodynamic status. The mean blood loss of GIII was only 539 ± 396 mL (vs. 5804 ± 8773 mL for GI and 718 ± 516 mL for GII). Hb at the end of the operation were 9.1 ± 1.5, 10.1 ± 1.6 and 11.4 ±1.8 g/dL for GI, GII and GIII respectively. It indicated no under- or over-transfusion in GI. The most important characteristic of GIII was minimal blood loss; it was not the result, but the cause by which avoidance of colloid and blood products were possible as evidenced by the fact that Hb at the end of the operation was still 11.4 ± 1.8 g/dL, despite GIII having had preoperative thrombocytopenia (Table 1). At present, there are no well-established preoperative indicators, including coagulation profiles, that may predict which LDLT recipients are suitable to receive crystalloids only. In a liver transplantation setting, it was indeed difficult to identify any specific risk factors; neither pre-operative nor intra-operative clotting parameters could predict the amount of intraoperative bleeding or blood products requirement [12,18]. From what we had learned from the 3.1% of our patients in the LT program, the fluid management should be individualized according to blood loss and hemodynamic stability. Crystalloids only provided not just intraoperative stable hemodynamics with less metabolic acidosis and less risk of IH, it had also reduced the one-year acute rejection rate of the liver graft in comparison to GII. The reason why 5% albumin increases the rate of acute rejection is not clear; it is likely that 5% albumin modulates the immunology of the recipient through binding of the immunosuppressive drugs and inducing immunosuppressive effect [5,21]. Crystalloids only (GIII) provided an excellent, 100% one- and three-year survival rate in our study. 

## 5. Conclusions

If the recipient did not require blood or albumin transfusions and could be managed by only crystalloid, then these were much fitter recipients who underwent a straightforward transplant without major blood loss and were therefore probably hemodynamically stable throughout the surgery. Therefore, these patients were much more likely to do well as surgery was straightforward. The outcome is better with significantly less one-year acute rejection postoperatively and had 100% one- and three-year survival rate. 

## Figures and Tables

**Table 1:** Patient characteristic and intraoperative parameters among groups.

	GI (*n* = 389)	GII (*n* = 73)	GIII (*n* = 15)	*p*
Age (Y)	53.6 ± 7.4	52.0 ± 9.8	52.5 ± 7.7	n.s.
Weight (Kg)	66.2 ± 11.0	69.3 ± 9.1	69.8 ± 13.5	n.s.
Anesthesia time (h)	14.1 ± 1.8	13.8 ± 1.3	13.1 ± 1.0	n.s.
Ascites (mL)	3176 ± 4926	289 ± 1191	10 ± 28.0	*#
Blood loss mixed with ascites (mL)	5804 ± 8773	718 ± 516	539 ± 396	*#
Red blood cell (mL)	4350 ± 6230	000	000	*#
FFP (mL)	1007 ± 1354	000	000	*#
Platelet (mL)	180.5 ± 301.1	000	000	*#
5% albumin (mL)	3480 ± 2379	1613 ± 780	000	*#+
Crystalloids (mL)	12652 ± 8192	9391 ± 9776	9580 ± 2257	*#
Hemoglobin pre-op (g/dL)	10.2 ± 1.9	13.2 ± 2.0	12.7 ± 1.8	*#
Hemoglobin end op (g/dL)	9.1 ± 1.5	10.1 ± 1.6	11.4 ± 1.8	*#+
Platelet count	68.3 ± 42.5	103.3 ± 54.8	54.9 ± 31.9	#+
INR	1.39 ± 0.5	1.11 ± 0.12	1.13 ± 1.1	n.s.
APTT	37.8 ± 11.1	33.0 ± 6.0	31.7 ± 4.4	n.s.
Serum albumin	2.8 ± 0.5	3.6 ± 0.6	3.5 ± 0.5	*#
Ca^++^ after Induction (mmol/L)	0.96 ± 0.49	0.90 ± 0.2	0.99 ± 0.11	n.s.
Ca^++^ dissection (mmol/L)	0.91 ± 0.18	0.88 ± 0.19	0.93 ± 0.11	n.s.
Ca^++^ anhepatic (mmol/L)	0.92 ± 0.2	0.93 ± 0.17	0.88 ± 0.11	n.s.
Ca^++^ reperfusion (mmol/L)	1.0 ± 0.21	1.07 ± 0.22	0.92 ± 0.15	n.s.
Ca^++^ end op (mmol/L)	1.0 ± 0.21	1.04 ± 0.21	0.92 ± 0.11	n.s.
Calcium Supplementation (mEq)	52.1 ± 59.2	28.0 ± 11.4	9.15 ± 6.3	*#+
pH after anesthesia	7.43 ± 0.5	7.43 ± 0.5	7.41 ± 0.6	#
pH during dissection	7.36 ± 0.6	7.39 ± 0.6	7.38 ± 0.6	#
pH anhepatic	7.27 ± 0.2	7.35 ± 0.4	7.35 ± 0.4	*#
pH reperfusion	7.27 ± 0.6	7.30 ± 0.4	7.32 ± 0.3	*#
pH end op	7.3 ± 0.7	7.32 ± 0.6	7.34 ± 0.5	n.s.
NaHCO_3_ (mEq)	317 ± 333	151 ± 9.4	113 ± 47.6	*#
Ventilation time (h)	39 ± 73.9	13.3 ± 7.7	16.5 ± 6.8	*#

n.s. = non-significance; * = *p* < 0.05 GI vs. GIII; + = *p* < 0.05 GII vs. GIII; # = *p* < 0.05 GI vs. GII.

**Table 2:** shows one- and three-year survival rate and one-year acute rejection amongst groups.

	GI (389)	GII (73)	GIII (15)	*p*-Value
One year acute rejection	20.4%	38.4%	14.3%	0.05
One year survival	94.1%	98.6%	100%	0.82
Three year survival	90.5%	94.5%	100%	0.127
